# Nutritional deficiencies among adults with beta thalassemia major in Vietnam

**DOI:** 10.1017/S1368980025101602

**Published:** 2025-12-26

**Authors:** Thi Ngoc Anh Hoang, Ha Linh Quach, Duc Binh Vu, Thi Thu Ha Nguyen, Khanh Linh Nguyen, Thi Thu Trang Dinh, Thi Mai An Bui

**Affiliations:** 1 Faculty of Public Health, PHENIKAA University, Hanoi 100000, Vietnam; 2 Prevention Research Collaboration, School of Public Health, The University of Sydney, Sydney, NSW, Australia; 3 National Institute of Hematology and Blood Transfusion, Hanoi 100000, Vietnam; 4 Hospital of Post and Telecommunications, Hanoi 100000, Vietnam; 5 Faculty of Medical Technology, PHENIKAA University, Hanoi 100000, Vietnam

**Keywords:** Beta thalassemia major, Nutritional status, Nutritional deficiencies, Vietnam

## Abstract

**Objective:**

To evaluate the nutritional status of adults with beta thalassemia major in Vietnam by analysing body composition parameters and assessing the adequacy of energy, macronutrient and micronutrient intake.

**Design:**

A cross-sectional study was conducted among adult patients with beta thalassemia major. Nutritional status was assessed using three components: BMI, body composition and dietary intake.

**Setting:**

Department of Thalassemia, The National Institute of Hematology and Blood Transfusion in Vietnam.

**Participants:**

317 adult patients with beta thalassemia major (54·2 % females, median age 32).

**Results:**

Nearly half (49·5 %) of the patients had a normal BMI, while 18·3 % were severely underweight and 32·2 % were underweight. Severe underweight patients exhibited lower body fat, bone mineral content and visceral fat. Average daily energy intake (1449·9 kcal) was significantly below the estimated requirement (2079·5 kcal), with 81·4 % of patients consuming less than 85 % of their energy needs. Severely underweight patients consumed 12·06 g/d less fat (95 % CI: 6·85, 17·26) and 10·42 g/d less protein (95 % CI: 4·42, 16·42) than normal weight patients. Severe deficiencies in Ca, Mg, Fe and B-complex vitamins were prevalent, with the lowest mean probability of adequacy for minerals and vitamins observed in severely underweight patients.

**Conclusion:**

This study provides the first comprehensive assessment of the nutritional status of beta thalassemia major patients in Vietnam, revealing critical gaps in energy and nutrient intake. Evidence-based strategies, including dietary education and interventions tailored to the unique needs of this population, are urgently needed to improve nutritional outcomes and overall health.

Beta thalassemia is a hereditary blood disorder characterised by reduced production of beta-globin, an Fe-rich protein in red blood cells^(1)^. Beta thalassemia major, also known as Cooley’s anaemia, represents the most severe form of transfusion-dependent anaemia within this spectrum^(2)^. Globally, its prevalence varies significantly by region, with highest rates observed in the Mediterranean, Asian Indian and Southeast Asian populations^(3)^.

On another stream of literature, nutritional status is a critical factor in managing beta thalassemia major, as patients face significant risks of malnutrition due to treatment protocols, especially the chronic effects of blood transfusions^(4)^. Frequent transfusions lead to Fe overload, which disrupts the metabolism of essential nutrients such as vitamin D, Ca, Mg, Zn and Fe, further exacerbating nutritional deficiencies^(5,6)^. These deficiencies, in turn, contribute to impaired growth, muscle wasting, weakened immune function and compromised body composition, including reduced lean mass and bone mineral density^(7,8)^. Maintaining adequate nutrition is therefore central not only to improve health outcomes and quality of life but also to reduce long-term complications in these patients.

Although several studies have examined the nutritional status of patients with beta thalassemia major globally^(9,10)^, evidence on the nutritional status of adults with beta thalassemia in Vietnam remains limited. In this country, beta thalassemia poses a significant public health challenge, with an estimated 10–15 % of the population carrying the thalassemia gene^(11)^. Most patients are of ethnic minority and/or residing in rural provinces, economic hardship, food insecurity and limited dietary diversity further increase their vulnerability to malnutrition^(12)^. In addition, dietary restrictions for patients with beta thalassemia, such as avoiding Fe-rich foods, can be challenging for patients to maintain a balanced and nutritious diet.

Previous research in Vietnam has largely focused on the genetics and clinical management of beta thalassemia, leaving a critical knowledge gap in understanding the nutritional challenges faced by this vulnerable population. By assessing body composition as well as energy, macronutrient and micronutrient intakes, this study addresses an urgent need for evidence to inform tailored nutrition interventions. Such evidence can help building suitable clinical and nutritional strategies and ultimately enhance the quality of life for patients with beta thalassemia major in Vietnam.

## Methods

### Study design

This is a cross-sectional study among adult patients diagnosed with beta thalassemia major at the Department of Thalassemia, the National Institute of Hematology and Blood Transfusion in Vietnam. National Institute of Hematology and Blood Transfusion is the largest institute in Vietnam dedicated to the treatment and research of blood-related disorders. Annually, National Institute of Hematology and Blood Transfusion provides treatment for approximately 1500 adults with beta thalassemia major. Participants are patients who were 18 years old or above at time of study, received regular treatment at National Institute of Hematology and Blood Transfusion and gave consent to join the study. Those with severe cardiomyopathy, severe liver disease or prolonged immobility were excluded. Participants were prospectively recruited from August to December 2024.

### Sample size

We used the Taro-Yamane formula for simple population survey sample^(13)^ as followed:

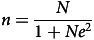




In this formula, *n* is the sample size, *N* is the population size and *e* is the level of significance. Based on an estimated population size of 1500 adults with beta thalassemia major treated annually at the institute and a significance level of 5 % (*e* = 0·05), the required sample size was calculated as 315.

To ensure adequate power, additional sample size calculation for continuous body-composition outcomes was conducted. Using an estimated sd of 9 % for percent body fat^(14)^ and a margin of error of 1 %, the required sample size was 315. Allowing for a 5 % attrition rate, a total of 317 eligible patients who met the study criteria were included in the final analysis.

### Data collection

Data collection tools are an online questionnaire developed on KoBoToolbox, a wall-mounted stadiometer and a TANITA InnerScan BC-541N electronic digital scale. Prior to data collection, the research team of four researchers underwent three training sessions to get familiar with the measuring tools, the questionnaire and related data collection procedures. The team approached patients directly in their wards and conducted data collection through face-to-face interviews. During the interview, the researcher read the questions out loud and logged the answer directly in the online survey with direct input from the patients. Total time of data collection per participant (which included completing the online questionnaire and using the scale) was about 30 min. During data curation, interviewers reported a non-response rate of approximately 3 %.

### Variables

#### Outcomes: patient’s nutritional status

Patients’ nutritional status was assessed through three components: BMI status, body composition and dietary intake.

#### BMI status

We calculated BMI using the formula: BMI = weight(kg)/height(m)^2^. Body weight and height were measured with participants barefoot and in light clothing. Height was recorded to the nearest 0·1 cm using a wall-mounted stadiometer, while weight was measured to the nearest 0·1 kg using a TANITA InnerScan BC-541N electronic digital scale. We used the Asia-Pacific BMI threshold to classify patients into five categories: Severely underweight (BMI < 16·5 kg/m^2^); underweight (BMI 16·5–18·4 kg/m^2^); normal weight (BMI 18·5–22·9 kg/m^2^); overweight (BMI 23·0–24·9 kg/m^2^) and obese (BMI ≥ 25·0 kg/m^2^). These thresholds are more appropriate for Asian populations due to differences in body composition and cardiometabolic risk compared with Western populations^(15–17)^.

#### Body composition

Body composition parameters included body fat percentage (%), bone mineral content (kg), total body water percentage (%), muscle mass (kg, %) and visceral fat rating. These measurements were assessed using bioelectrical impedance analysis with the TANITA InnerScan BC-541N electronic digital scale and recorded electronically. A single bipolar measurement was performed using two surface electrodes attached to the dorsal region of the foot. A low-density electrical current (50 kHz) was introduced, and tissue resistance was measured to calculate lean mass and fat tissue^(18)^.

Measurements were taken twice on two consecutive mornings after participants had urinated and while wearing light clothing without footwear. The final recorded results were the mean of the two measurements. If a discrepancy of ≥ 5 % was observed between the two measurements, a third measurement was taken the following morning using the same procedure. In such cases, the final recorded results were the mean of the two closest measurements.

#### Dietary intake

Dietary intake was assessed using 24-h dietary recalls over three consecutive days, excluding any celebrations meals or meals on special event days. Reported food items and portion sizes were converted into nutrient intakes using the 2007 Vietnamese Food Composition Table in Excel^(19)^, adjusted for nutrient retention of cooked foods^(20,21)^. For foods of which nutrient information was missing, we substituted nutrient intakes based on the food composition database from the Thai food composition table^(22)^ and the Asian food composition table^(23)^ instead. Each recalled food item was cross-referenced with the corresponding food composition entry, and nutrient values were calculated by multiplying the consumed amount of each food by its nutrient content per unit. Daily nutrient intakes were then summed across all foods for each participant. From these data, we derived analytical nutrition variables including total energy, macronutrients (carbohydrates, fats and proteins), minerals and vitamins.

#### Covariates

Covariates included socio-demographic characteristics (age, sex, ethnicity, residence, education level, marital status, employment status, individual monthly income, monthly medical expenses and any financial support received from the government or other social agencies) and health risk factors (years since beta thalassemia diagnosis, comorbidity and any smoking or alcohol consumption in the past 6 months). Participants were asked to estimate their average individual monthly income and monthly medical expenses over the past 3 months. The Healthy Eating Scale, a validated tool in Vietnam^(24)^, was used to measure the frequency of healthy food consumption. These covariates were chosen based on the literature on factors associated with the nutritional status of thalassemia patients^(6,14,25,26)^.

### Statistical analysis

Data were exported from KoBoToolbox to Excel for management and subsequently imported into Stata 18·0 for cleaning and analysis. First, we tabulated socio-demographic characteristics and health risk factors by patients’ BMI categories. Second, body composition parameters were summarised, stratified by sex and BMI categories and then compared against reference ranges for body composition in a healthy population^(27–31)^. Descriptive measures included means and sd for normally distributed continuous variables (bone mineral content, muscle mass value and muscle mass), medians with interquartile ranges (IQR) for skewed continuous variables (age, individual income per month, medical expenditure per month, duration of beta thalassemia, healthy eating score, body fat, total body water and visceral fat rating) and frequencies with percentages for categorical variables. Between-group differences were assessed using the one-way ANOVA or Kruskal–Wallis test for continuous variables and Pearson’s *χ* ^2^ test or Fisher’s exact test for categorical variables.

Third, we summarised energy and macronutrient consumption by patients’ BMI categories. Assuming that patients with beta thalassemia major generally exhibit low physical activity levels, we calculated their energy expenditure requirement (EER) using the formula for low-active individuals^(32)^: EER = 753·07 – (10·83 × age) + (6·50 × height) + (14·10 × weight). We then calculated the percentage of patients who consumed at least 85 % of their EER based on their actual daily energy intake^(33)^. The percentage of energy from macronutrient intakes (carbohydrates, fat and protein) was compared with the acceptable macronutrient distribution ranges^(34)^ for assessing percentage of insufficient or excessive intake in each component. Following the recommendation of the U.S. Institute of Medicine^(35)^, the acceptable macronutrient distribution ranges for protein, fat and carbohydrates are 10–35 %, 20–35 % and 45–65 %, respectively. Next, we also summarised minerals and vitamins intakes by BMI categories. We calculated the probability of adequacy consumption based on thresholds outlined in Vietnam’s national dietary guideline^(19)^ (see online supplementary material, Supplemental Table 1s). The mean probability of adequacy (MPA) for vitamins and minerals was computed and reported. Between-group differences in macronutrient and micronutrient intakes were assessed using one-way ANOVA or the Kruskal–Wallis test, depending on distributional assumptions.

Lastly, we employed linear regression models to evaluate the relationship between BMI categories and (i) macronutrient intakes (carbohydrates, fat and protein) and (ii) the MPA for vitamins and minerals. Both univariate and multivariable models were adjusted for socio-demographic characteristics and health risk factors listed above. Model assumptions were evaluated using the Shapiro–Wilk test, Breusch–Pagan test, residual-*v*.-fitted plots and Q-Q plots. Although formal tests indicated departures from normality and homoscedasticity, visual inspections suggested acceptable model fit. To address potential violations, all models were estimated with heteroskedasticity-robust (Huber-White) standard errors. Results were presented as mean differences and 95 % CI.

## Results

Among 317 patients with beta thalassemia major, nearly half (49·5 %, *n* 157) had a normal weight, 18·3 % (*n* 58) were classified as severely underweight and 32·2 % (*n* 102) were underweight (Table 1). No patients were classified as overweight or obese.

**Table 1.** Characteristics of patients with beta thalassemia major, stratified by BMI status

	Total (*n* 317)		Severely underweight (*n* 58, 18·3 %)		Underweight (*n* 102, 32·2 %)		Normal weight (*n* 157, 49·5 %)		*P*-value^*^
	*n*	%	*n*	%	*n*	%	*n*	%	
Socio-demographic									
Age									
Median	32		31		33		33		0·61
IQR	24–41		22–39		24–44		25–40		
Female	172	54·3	22	37·9	44	43·1	106	67·5	< 0·001
Ethnicity									0·20
Kinh	119	37·5	28	48·3	37	36·3	54	34·4	
Thai	79	24·9	15	25·9	20	19·6	44	28·0	
Muong	64	20·2	9	15·5	26	25·5	29	18·5	
Tay	28	8·8	3	5·2	7	6·9	18	11·5	
Others	27	8·5	3	5·2	12	11·8	12	7·6	
Residence									0·95
Rural	238	75·1	43	74·1	77	75·5	118	75·2	
Urban	56	17·7	11	19·0	19	18·6	26	16·6	
Semi-urban	23	7·3	4	6·9	6	5·9	13	8·3	
Education									0·50
Illiterate	14	4·4	1	1·7	5	4·9	8	5·1	
Primary school	40	12·6	9	15·5	15	14·7	16	10·2	
Lower secondary school	117	36·9	27	46·6	38	37·3	52	33·1	
Higher secondary school	106	33·4	15	25·9	31	30·4	60	38·2	
College/University or higher	40	12·6	6	10·3	13	12·7	21	13·4	
Marital status									0·006
Never married	176	55·5	43	74·1	60	58·8	73	46·5	
Living with Partner/Spouse	125	39·4	14	24·1	36	35·3	75	47·8	
Divorced	16	5·0	1	1·7	6	5·9	9	5·7	
Employment status									0·39
Employed	30	9·5	6	10·3	7	6·9	17	10·8	
Self-employed	122	38·5	17	29·3	40	39·2	65	41·4	
Unemployed	165	52·1	35	60·3	55	53·9	75	47·8	
Individual income per month (USD)									
Median	0		0		0		78·6		0·042
IQR	0–196·4		0–78·6		0–196·4		0–235·7		
Medical expenditure per month (USD)									
Median	78·6		98·2		78·6		78·6		0·73
IQR	47·1–117·9		58·9–117·9		39·3–117·9		58·9–117·9		
Received financial support	266	83·9	52	89·7	90	88·2	124	79·0	0·059
Health risks									
Duration of beta thalassemia (years)									
Median	24·0		28·0		25·0		22·0		0·007
IQR	17·0–33·0		19·0–34·0		17·0–35·0		14·0–30·0		
With comorbidity	104	32·8	18	31·0	36	35·3	50	31·8	0·80
Smoking in the last 6 months	35	11·0	9	15·5	13	12·7	13	8·3	0·26
Drinking in the last 6 months	89	28·1	18	31·0	32	31·4	39	24·8	0·45
Healthy eating score									
Median	8·0		7·0		8·0		8·0		0·27
IQR	6·0–9·0		6·0–9·0		6·0–9·0		6·0–10·0		

IQR, interquartile range.

* *P* values were calculated using one-way ANOVA, Kruskal–Wallis test, Pearson’s *χ* ^2^ test and Fisher’s exact test.

The median age of all patients was 32 years old (IQR: 24–41). Over half of patients were female (54·3 %, *n* 172), with a higher proportion of females as BMI increased (severely underweight: 37·9 %, underweight: 43·1 %, normal weight: 67·5 %, *P*-value < 0·001).

Over one-third of patients identified as Kinh, the major ethnicity in Vietnam (37·5 %), while the remaining patients belonged to other ethnicities, such as Thai (24·9 %), Muong (20·2 %) and Tay (8·8 %). Three-fourths of patients resided in rural areas. A small proportion had a college or university degree or higher (12·6 %), while a larger group completed lower secondary school (36·9 %) and higher secondary school (33·4 %).

Over half of the patients never married (55·5 %) and were unemployed (52·1 %). The prevalence of patients who never married or unemployed was higher in the severely underweight (74·1 % and 60·3 %, respectively) and underweight (58·8 % and 53·9 %, respectively) groups compared with those of normal weight (46·5 % and 47·8 %, respectively), but the difference was only significant in marital status (*P* = 0·006).

The median monthly individual income of underweight or severely underweight patients (0 USD per month) was lower than those of normal weight patients (78·6 USD per month) (*P* = 0·042). Meanwhile, severely underweight patients reported the highest monthly medical expenses (median: 98·2 USD, IQR: 58·9–117·9 USD) and highest proportion to receive financial support (89·7 %), compared with underweight (medical expenses: 78·6 USD, 39·3 – 117·9; 88·2 % receiving financial support) and normal weight patients (medical expenses: 78·6 USD; 58·9–117·9; 79·0 % receiving financial support), but the differences were not statistically significant.

Patients with severely underweight status were diagnosed with beta thalassemia (median: 28 years; IQR: 19–34) for a longer time compared with those who were underweight (25 years; 17–35) and those of normal weight (22 years; 14–30) (*P* = 0·007). Underweight patients had the highest percentage of having comorbidity (35·3 %) compared with their counterparts, while severely underweight patients had a higher prevalence of smoking (15·5 %) and drinking (31 %) compared with patients with normal weight (8·3 % and 24·8 %, respectively). Additionally, severely underweight patients reported the lowest healthy eating score (median: 7; IQR: 6–9) compared with underweight patients (8; 6–9) and normal weight patients (8; 6–10).

Table 2 presents the body composition parameters of patients with beta thalassemia major, categorised by BMI status and sex, in comparison to reference ranges. Overall, severely underweight patients demonstrated a significant lower percentage of body fat, bone mineral content and visceral fat rating, along with a higher percentage of muscle mass, compared with normal weight patients, regardless of sex.

**Table 2.** Body composition parameters of patients with beta thalassemia major, stratified by sex and BMI status

Body compositions	Males								Females							
	Severely underweight (*n* 36)		Underweight (*n* 58)		Normal weight (*n* 51)		*P* value^*^	Reference ranges	Severely underweight (*n* 22)		Underweight (*n* 44)		Normal weight (*n* 106)		*P*-value^*^	Reference ranges
Body fat, % median IQR	11·8	8·6, 14·8	13·6	9·1, 15·2	13·5	11·2, 17·5	0·015	14–17·9	21·1	18·3, 23·0	23·9	22·3, 24·8	27·5	25·7, 29·5	< 0·001	15–24·9
Bone mineral content, kg mean sd	1·9	0·2	2·2	0·2	2·4	0·2	< 0·001	2·5–3·5	1·5	0·2	1·7	0·2	1·9	0·3	< 0·001	1·5–2·5
Total body water, % median IQR	57·9	56·0, 60·1	57·5	55·8, 60·4	57·9	55·8, 60·0	0·80	50–65	54·2	51·4, 56·0	52·7	51·0, 53·8	50·0	48·7, 51·7	< 0·001	45–60
Muscle mass value, kg mean sd	34·3	4·3	39·9	4·3	43·0	4·3	< 0·001	NA	28·1	1·4	30·6	2·3	33·4	3·2	< 0·001	NA
Muscle mass, % mean sd	84·3	5·1	83·0	3·8	81·4	3·9	0·008	73–86	75·3	3·3	72·5	1·9	68·1	3·0	< 0·001	62–73·5
Visceral fat rating, median IQR	1·0	1·0, 1·0	1·0	1·0, 2·5	3·0	1·0, 5·5	< 0·001	1–12	1·0	1·0, 1·0	1·0	1·0, 2·0	3·0	2·5, 4·0	< 0·001	1–12

NA, not applicable; IQR, inter-quartile range.

* *P* values were calculated using one-way ANOVA or Kruskal–Wallis test.

Among male patients, severely underweight individuals had significantly lower values for body fat (median 11·8 % *v*. 13·6 % in underweight and 13·5 % in normal weight; *P* = 0·015), bone mineral content (1·9 kg *v*. 2·2 kg in underweight and 2·4 kg in normal weight; *P* < 0·001), muscle mass value (34·3 kg *v*. 39·9 kg in underweight and 43·0 kg in normal weight; *P* < 0·001) compared with underweight and normal-weight ones. Except for body fat and bone mineral content, all body composition metrics for male patients fell within the reference ranges reported for healthy adults^(27–31)^.

In contrast, female patients who were severely underweight exhibited significantly lower values for body fat (21·1 % *v*. 23·9 % in underweight and 27·5 % in normal weight; *P* < 0·001), bone mineral content (1·5 kg *v*. 1·7 kg in underweight and 1·9 kg in normal weight; *P* < 0·001) and muscle mass value (28·1 kg *v*. 30·6 kg in underweight and 33·4 kg in normal weight; *P* < 0·001) compared with underweight and normal-weight females. Normal weight female patients had the lowest total body water (50·0 % *v*. 52·7 % in underweight and 54·2 % in severely underweight; *P* < 0·001) and muscle mass percentage (68·1 % *v*. 72·5 % in underweight and 75·3 % in severely underweight; *P* < 0·001) among three categories of BMI status. Compared with reference values^(27–31)^, normal weight female patients had a higher body fat percentage, while severely underweight female patients had a higher muscle mass percentage.

Table 3 shows energy and macronutrient consumption of patients with beta thalassemia major, stratified by BMI status. The total mean expected EER for all patients was 2079·5 kcal/d, which was notably higher than the average daily energy intake of 1499·9 kcal/d. Average energy intake significantly increased with BMI status (1389·9 kcal in severely underweight patients; 1485·9 kcal in underweight patients and 1549·6 kcal in normal weight patients; *P* = 0·016). About 86 % of severely underweight patients consumed less than 85 % of their EER, compared with 83·3 % among underweight patients and 78·3 % among normal weight patients.

**Table 3.** Energy and macronutrient consumption of patients with beta thalassemia major, stratified by BMI status

	Total (*n* 317)		Severely underweight (*n* 58)		Underweight (*n* 102)		Normal weight (*n* 157)		*P*-value^*^
Energy, kcal/d									
EER, kcal^†^ mean sd	2079·5	185·5	1991·9	193·8	2063·0	200·6	2122·6	158·2	< 0·001
Energy intake, kcal mean sd	1499·9	368·1	1389·9	267·6	1485·9	260·9	1549·6	444·0	0·016
< 85 % EER, %	81·4		86·2		83·3		78·3		0·35
Carbohydrate									
Amount consumed, g	199·6	57·4	194·2	50·9	204·8	60·4	198·2	57·8	0·49
% energy, mean sd	53·8	11·1	56·4	12·0	55·3	13·3	51·9	8·5	0·008
Inadequate consumption^‡^, %	17·7		12·1		13·7		22·3		0·098
Excessive consumption, %	8·5		13·8		13·7		3·2		0·003
Fat									
Amount consumed, g	49·0	19·8	41·0	14·9	47·1	16·0	53·1	22·4	< 0·001
% energy, mean sd	29·3	8·6	26·2	6·8	28·4	7·9	31·0	9·2	< 0·001
Inadequate consumption^‡^, %	11·4		17·2		13·7		7·6		0·095
Excessive consumption, %	22·4		10·3		21·6		27·4		0·028
Protein									
Amount consumed, g	72·3	22·2	64·6	17·1	72·4	17·2	75·0	25·8	0·009
% energy, mean sd	19·3	3·6	18·5	3·1	19·7	4·1	19·4	3·5	0·17
Inadequate consumption^‡^, %	0		0		0		0		–
Excessive consumption, %	0		0		0		0		–

* The *P* value was calculated using one-way ANOVA tests or Kruskal–Wallis tests.

† EER: energy expenditure requirement was calculated based on the equation: = 753·07 – (10·83 × age) + (6·50 × height) + (14·10 × weight).

‡ Insufficient and excessive intake levels based on the acceptable macronutrient distribution ranges (AMDRs): protein 10–35 %; fat 20–35 % and carbohydrate 45–65 % of total energy.

The mean carbohydrate intake across all patients was 199·6 g/d, contributing to 53·8 % of total energy intake. Carbohydrate intake among severely underweight patients was accounted for the highest percentage of their energy consumption (56·4 %), compared with underweight (55·3 %) and normal weight patients (51·9 %) (*P*-value = 0·008). Among the three BMI categories, severely underweight patients had the highest percentage of excessive carbohydrate consumption (13·8 % *v*. 13·7 % in underweight and 3·2 % in normal weight; *P* value = 0·003).

The mean fat intake among all patients was 49 g/d, accounting for 29·3 % of total energy intake. Severely underweight patients consumed the least fat (41 g/d, 26·2 % of energy intake), followed by underweight patients (47·1 g/d, 28·4 % of energy intake) and normal weight patients (53·1 g/d, 31 % of energy intake) (*P* value < 0·001). Severely underweight patients also had the highest prevalence of inadequate fat consumption (17·2 %) compared with underweight patients (13·7 %) and normal weight patients (7·6 %) (*P* value = 0·095).

Protein intake averaged 72·3 g/d among all patients, contributing to 19·3 % of total energy intake. All patients, regardless of BMI status, met adequate protein intake levels, with no cases of inadequate or excessive protein consumption.

**Figure 1.**
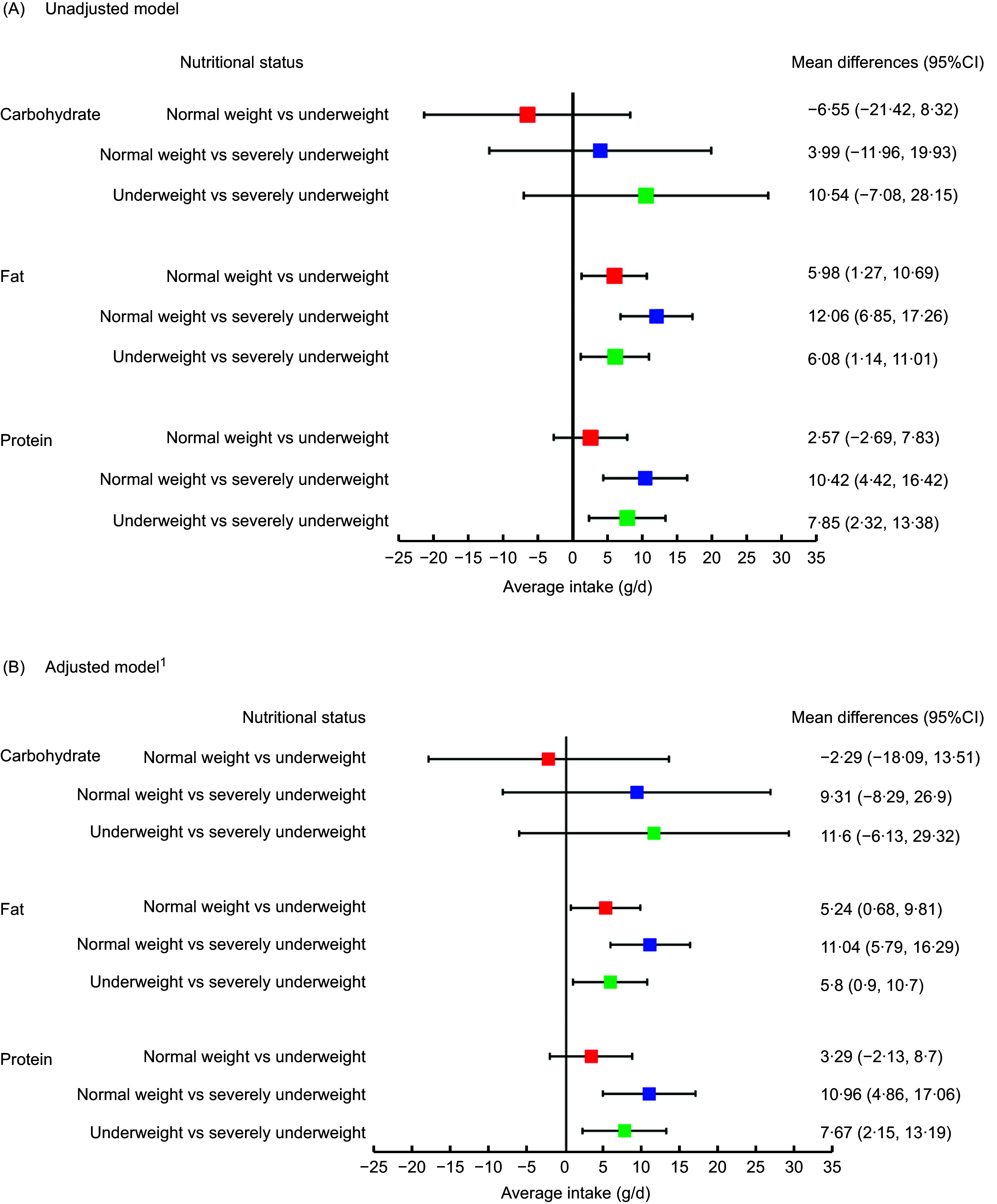
Mean differences in macronutrient intake of patients with beta thalassemia major across BMI status: results from univariable (Panel A) and multivariable regression models (Panel B).

Figure 1 illustrates the mean differences in three macronutrients intakes (carbohydrate, fat and protein) between the BMI categories of patients with beta thalassemia major, resulting from univariate (Panel A) and multivariable linear regression models (Panel B).

In panel A, there were no significant differences observed in carbohydrate intake across three BMI categories. In terms of fat intake, severely underweight patients consumed 12·06 g/d less fat (95 % CI: 6·85, 17·26) than normal weight patients and 6·08 g/d less fat (1·14, 11·01) than underweight patients, while underweight patients consumed 5·98 g/d less fat (95 % CI: 1·27, 10·69) than their normal weight counterparts. For protein intake, severely underweight patients consumed 10·42 g/d less protein (95 % CI: 4·42, 16·42) than normal weight patients and 7·85 g/d less protein (95 % CI: 2·32, 13·38) than underweight patients.

In panel B, after adjusting for socio-demographic and health risk variables, the significant differences remained unchanged.

Table 4 presents the probability of adequacy for mineral and vitamin intake among patients with beta thalassemia major, stratified by BMI status. Median intakes for minerals and vitamins across BMI categories can be found in online supplementary material, Supplemental Table 2s.

**Table 4.** Probability of adequacy for mineral and vitamin intake by BMI status

Probability of adequacy	Total (*n* 317)	Severely underweight (*n* 58)	Underweight (*n* 102)	Normal weight (*n* 157)	*P*-value^*^
	%				
Minerals					
Ca	1·9	3·4	2·0	1·3	0·58
Phosphorus	75·7	67·2	75·5	79	0·20
Mg	4·1	1·7	4·9	4·5	0·59
Fe	8·8	5·2	10·8	8·9	0·48
Zn	37·2	31	34·3	41·4	0·29
Selen	94·6	98·3	93·1	94·3	0·37
Cu	32·5	29·3	31·4	34·4	0·75
Vitamins					
Vitamin A, RAE	66·2	50	66·7	72	0·010
Vitamin D	21·1	20·7	23·5	19·7	0·76
Vitamin E	37·2	37·9	32·4	40·1	0·45
Vitamin K	25·2	15·5	26·5	28	0·16
Vitamin C	17·7	6·9	16·7	22·3	0·030
Thiamine	28·1	22·4	24·5	32·5	0·22
Riboflavin	3·8	3·4	2·9	4·5	0·81
Niacin	22·7	15·5	21·6	26·1	0·24
Acid pantothenic	13·6	10·3	15·7	13·4	0·63
Vitamin B_6_	14·2	13·8	15·7	13·4	0·87
Biotin	1·3	3·4	0	1·3	0·17
Folate	5·7	6·9	7·8	3·8	0·36
Vitamin B_12_	16·1	25·9	15·7	12·7	0·067
MPA	26·4	23·4	26·1	27·7	0·13

MPA, mean probability of adequacy; RAE, retinol activity equivalent.

* *P* value was calculated using one-way ANOVA tests or Kruskal–Wallis tests.

For mineral intake, a very low percentage of patients consumed adequate amounts of Ca (1·9 %), Mg (4·1 %) and Fe (8·8 %). Approximately one-third of patients met the recommended daily intake for Zn (37·2 %) and Cu (32·5 %), while the majority consumed adequate amounts of phosphorus (75·7 %) and Se (94·6 %). Among the three BMI categories, severely underweight patients had lower mineral intakes compared with underweight and normal weight patients, although these differences were not statistically significant.

For vitamin intake, the prevalence of adequate intake of fat-soluble vitamins was highest in vitamin A consumption (66·2 %) and lowest in vitamin D consumption (21·1 %). Significant differences in sufficient vitamin consumption among BMI categories were observed for vitamins A and C. While 50 % of severely underweight patients consumed adequate amounts of vitamin A, the proportions were higher among underweight (66·7 %) and normal weight patients (72 %) (*P*-value = 0·010). Similarly, only 6·9 % of severely underweight patients met the adequate intake for vitamin C, compared with 16·7 % of underweight patients and 22·3 % of normal weight patients (*P* value = 0·030). Meanwhile, the adequacy of B-complex water-soluble vitamins was notably low, with only 1·3 % of patients consuming sufficient biotin, 3·8 % for riboflavin and 5·7 % for folate. There were no statistically significant differences between proportion of adequate consumptions in these vitamins between three BMI categories.

The MPA for minerals and vitamins was 26·4 %, with the lowest value observed in severely underweight patients (23·4 %) and the highest in normal weight patients (27·7 %), although the difference was not statistically significant (*P* value = 0·13).

**Figure 2.**
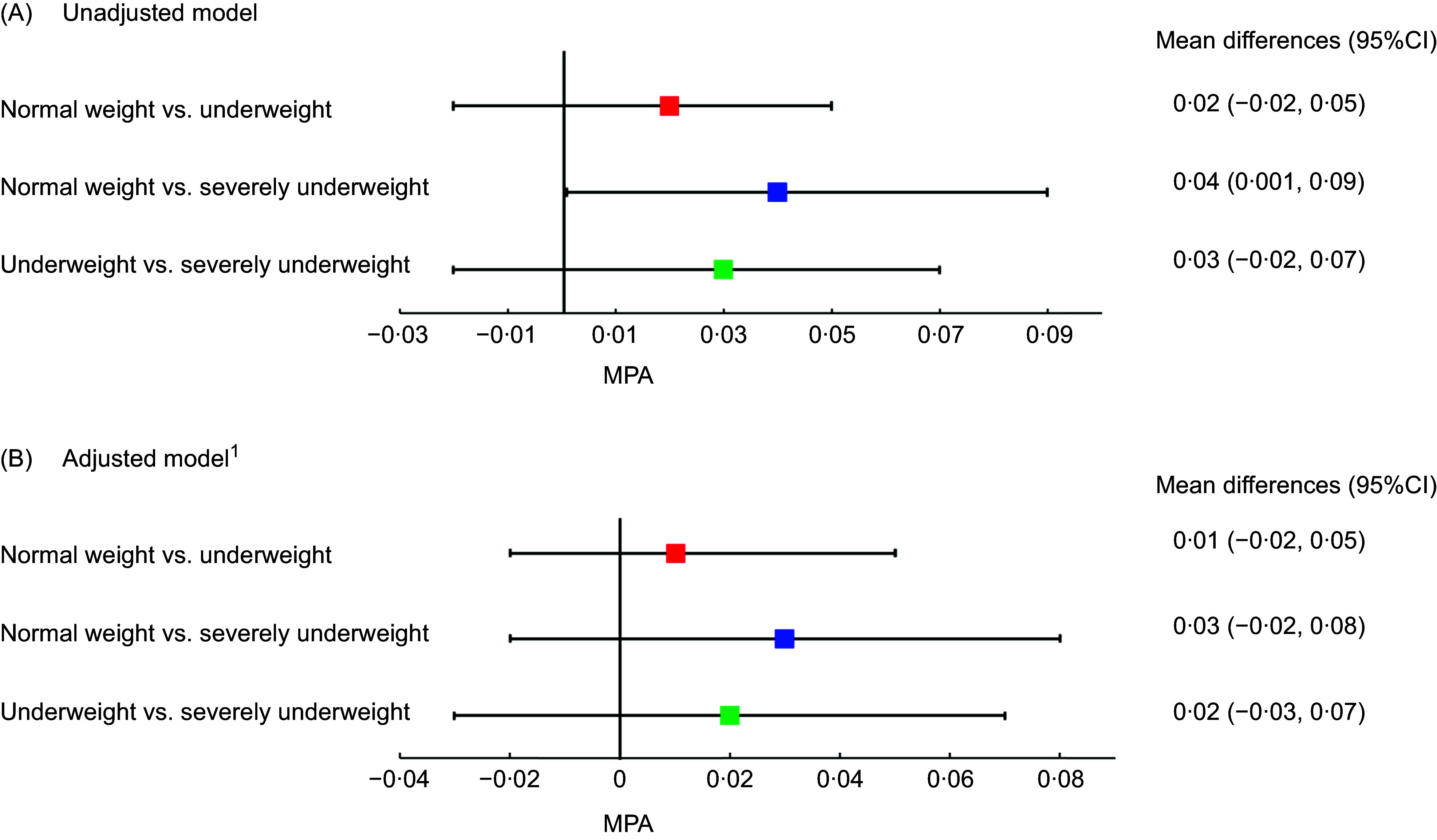
Mean probability of adequacy of minerals and vitamins intakes of patients with beta thalassemia major across BMI status: results from univariable (Panel A) and multivariable regression (Panel B).

Figure 2 presents the MPA for mineral and vitamin intakes of patients with beta thalassemia major across BMI categories. Panels A and B display findings from the univariable and multivariable linear regression models, respectively. In Panel A, severely underweight patients had a significantly lower MPA compared with normal weight patients (mean difference: 0·04, 95 % CI: 0·001, 0·09). However, this association weakened and lost statistical significance in Panel B. No significant differences in MPA were observed between normal weight and underweight patients, nor between underweight and severely underweight patients, in both models.

## Discussion

To our knowledge, this was the first study to assess the nutritional status of patients with beta thalassemia major in Vietnam. We found a high prevalence of undernutrition among patients with beta thalassemia major, with over half being underweight or severely underweight. Severely underweight patients exhibited significantly lower body fat in males and reduced bone mineral content in both males and females compared with their normal weight counterpart. Additionally, severely underweight patients consumed significantly lower fat, protein, minerals and vitamins, compared with normal weight patients.

Our results indicate that more than half of the patients did not fall within the normal weight BMI category, with 18·3 % classified as severely underweight and 32·2 % as underweight. These proportions are notably higher compared with previous studies. For instance, earlier research reported a prevalence of 7·5 % underweight among beta thalassemia major patients in Greece^(14)^ and 5·2 % among transfused thalassemia patients in North America. The high prevalence of poor nutritional status in our study may be attributed to several socio-demographic factors, including a high proportion of patients with lower education levels, unemployment and low monthly income. Socio-economic status plays a critical role in shaping dietary habits and other health behaviours^(36)^. Patients with beta thalassemia major often require frequent hospital visits for medical procedures and generally have poorer physical health than the general population^(37)^, which may limit their ability to achieve higher socio-economic status and, in turn, hinder their access to a healthier diet.

Additionally, a large proportion of patients in this study resided in rural areas and belonged to ethnic minority groups. Despite Vietnam’s rapid modernisation and urbanisation, many rural and ethnic minority populations continue to face economic and educational challenges^(38)^. Malnutrition and limited nutritional knowledge remain disproportionately prevalent in these communities^(39)^, potentially influencing the dietary patterns of patients with beta thalassemia major. Furthermore, smoking and alcohol consumption were more common in the severely underweight group, which could further compromise healthy eating habits^(40)^. While this study did not explore additional factors associated with poor nutritional status, we recommend further research to investigate disparities in socio-demographic characteristics, dietary habits and other health-related behaviours among patients with beta thalassemia major.

Our findings indicate that severely underweight patients consumed significantly lower amounts of fat and protein compared with underweight and normal weight individuals, even after adjusting for socio-demographic factors and health risk behaviours. This can be explained that fat and protein are critical for maintaining energy balance, muscle mass and overall body composition. Deficiencies in these macronutrients can exacerbate weight loss and compromise immune function^(41,42)^, thus leading to undernutrition in this population. These results highlight the need for targeted nutritional interventions, such as protein- and energy-dense dietary, particularly for patients with severe underweight, to prevent further deterioration of nutritional status and associated health complications.

Severe micronutrient deficiencies were also evident, with fewer than 10 % of participants meeting adequate intake levels for essential minerals such as Ca, Mg and Fe, as well as B-complex vitamins such as biotin, riboflavin and folate, according to Vietnam’s national dietary guideline^(19)^. Dietary adequacy was poorest among severely underweight patients, as reflected in the lowest mean probability of adequacy for both mineral and vitamin intake, particularly vitamins A and C, compared with underweight and normal-weight patients. However, significant deficiencies in energy, vitamins and minerals were observed across all patients in this study.

While this finding aligns with previous research on beta thalassemia patients^(4,10,14)^, it may also reflect dietary habits in Vietnam, where rice and rice-based products predominate. A previous study in China reported that the percentage of energy from carbohydrates was significantly higher among underweight individuals compared with those with normal weight^(43)^. Similarly, our study found that carbohydrate intake among severely underweight patients accounted for the highest percentage of their total energy consumption, compared with both underweight and normal weight patients. This can be explained by cultural practice in Vietnam and other Asian countries where rice and rice-based products are expected in every meal, and rice is the main agricultural products. However, for patients with beta thalassemia – who are already vulnerable to nutritional deficiency and malnutrition, over-reliance on rice products without diversification for other foods may limit intake of other essential nutrients. This couples with dietary restrictions for patients with beta thalassemia may further limit their food variety and nutrient intake^(44)^, contributing to poor dietary diversity.

Our findings also indicate a disproportionately high prevalence of underweight and severely underweight status among male patients, consistent with current literature^(8)^. This observation raises important questions about potential sex-specific vulnerability to undernutrition in patients with beta thalassemia major. In Vietnam, societal norms may influence food distribution within households, with men often prioritising physical labour or other responsibilities, potentially neglecting their dietary needs^(45)^. Additionally, studies suggest that men are generally less likely than women to seek medical advice or adhere to dietary recommendations, further exacerbating their risk of undernutrition^(46,47)^.

Our analysis revealed significant imbalances in body composition, including a low-fat percentage in male patients and reduced bone mineral content in both sexes, even among those with a normal BMI. These imbalances may be linked to multiple factors associated with beta thalassemia major, including reduced physical activity and frequent blood transfusions. Patients with beta thalassemia major often experience decreased physical activity due to severe anaemia and cardiomyopathies resulting from Fe overload^(48)^. Given the strong association between physical activity and body composition, reduced exercise can lead to alterations in fat distribution, including lower fat percentage and increased adipose tissue^(49)^. Additionally, Fe toxicity from repeated transfusions may impair the absorption of essential minerals and vitamins, such as Ca and vitamin D, further exacerbating bone mineral deficits and compromising bone health^(50)^. With a median disease duration of nearly 30 years, participants in this study were inevitably affected by cumulative blood transfusions and related medical complications, contributing to their suboptimal body composition. These findings emphasise the importance of regular body composition assessments in patients with beta thalassemia major to guide personalised dietary and lifestyle interventions aimed at improving overall health.

We acknowledge several limitations. First, we did not collect data on different types of carbohydrates and fats consumed, which would allow for more specific analysis of dietary component. Second, as this was a cross-sectional, single-centre study (albeit the largest centre for thalassemia treatment in Vietnam), the data may not be fully generalisable to all individuals with beta thalassemia major in Vietnam or in other settings. The self-reported nature of the study is also subjected to reporting bias. We emphasise the need for multi-centre studies that are representative of the broader population in Vietnam. Third, due to the lack of reference ranges for body composition in patients with beta thalassemia major, we used reference values from the healthy population, which may have led to an overestimation of the prevalence of inadequacy. Fourth, we could not collect 24-h dietary recalls on non-consecutive days due to short hospitalisation/visiting time among patients (about 3–5 d in the department) and limiting missing data. Fifth, the use of bioelectrical impedance analysis might be too sensitive to patient hydration status, which may lead to inaccurate estimates of body composition in individuals who are overhydrated or dehydrated at the time of measurement.

Despite these limitations, this study provides valuable insights into the dietary intake of patients with beta thalassemia major – a topic that remains largely underexplored in Vietnam and surrounding regions. This study was careful selection of participants, and the use of standardised measurement tools, including stadiometers and electronic scales, as well as structured questionnaires. These measures help ensure that the observed associations within our study population are reliable. While BMI is a widely used measure of nutritional status, it may not be sensitive enough to detect changes in lean and adipose tissue mass, potentially leading to misclassification. To address this, we utilised bioelectrical impedance analysis methods for a more accurate assessment of body composition in patients with beta thalassemia major. Overall, our findings enhance understanding of the nutritional challenges faced by this population.

Our findings underscore the importance of primary public health interventions to improve nutritional outcomes among patients with beta thalassemia major. In our study, patients with beta thalassemia major face with several social and economic challenges due to poor health status and inability to join employment market. These challenges constraints can limit their access to diverse, nutrient-rich foods for their nutritional requirements. Although beyond the scope of this study, these patients and their caregivers can benefit from health education and support from health system for their physical and nutritional needs as well. Therefore, hospital-based nutrition programmes and health education initiatives for patients and caregivers can help improve patients’ awareness and practice of healthy diet. These interventions should consider the prevalence of culturally and socially diverse population of patients with thalassemia, tailoring for lower income and ethnic minority individuals. Integrating such interventions into existing healthcare frameworks would help address structural barriers and ensure that nutritional support reaches the most vulnerable patients.

### Conclusion

This study offers the first comprehensive evaluation of the nutritional status of patients with beta thalassemia major in Vietnam, highlighting a high prevalence of undernutrition in this population. Significant deficiencies in energy intake, essential minerals and vitamins were observed, along with imbalances in body composition, including low fat percentage and reduced bone mineral content. Interventions should focus on enhancing both the quality and quantity of dietary intake to address the complex nutritional challenges faced by patients with beta thalassemia major.

## Supporting information

Hoang et al. supplementary material Hoang et al. supplementary material

## Supplementary material

For supplementary material accompanying this paper visit https://doi.org/10.1017/S1368980025101602
