# Supplementary material for: Nutritional deficiencies among adults with beta thalassemia major in Vietnam
Source: Public Health Nutr. 2025 Dec 26;29(1):e6. doi: 10.1017/S1368980025101602 (PMC12809608; doi:10.1017/S1368980025101602)
Supplement: Hoang et al. supplementary material [file S1368980025101602sup001.docx]

**Supplemental Table 1s. Recommended dietary allowance values used in calculating probability of adequacy of nutrient intakes^1^**

|  | Age groups | Male | Female |
| --- | --- | --- | --- |
| **Minerals** |  |  |  |
| Calcium (mg/day) | 18-19y | 1000 | 1000 |
|  | 20-49y | 800 | 800 |
|  | 50-69y | 800 | 900 |
| Magnesium (mg/day) | 18-19y | 350 | 300 |
|  | 20-29y | 340 | 270 |
|  | 30-49y | 370 | 290 |
|  | 50-69y | 350 | 290 |
| Phosphorus (mg/day) | 18-19y | 1250 | 1250 |
|  | 20-69y | 700 | 700 |
| Iron (mg/day) | 18-19y | 17.5 | 29.7 |
|  | 20-49y | 11.9 | 26.1 |
|  | 50-69y | 11.9 | 10.0 |
| Zinc (mg/day) | 18-69y | 10.0 | 8.0 |
| Selen (µg/day) | 18-19y | 32.0 | 26.0 |
|  | 20-69y | 34.0 | 26.0 |
| Copper (µg/day) | 18-19y | 890 | 890 |
|  | 20-69y | 900 | 900 |
| **Vitamins** |  |  |  |
| Vitamin A (µg/day) RAE | 18-29y | 850 | 650 |
|  | 30-49y | 900 | 700 |
|  | 50-69y | 850 | 700 |
| Vitamin D (µg/day) | 18-49y | 15 | 15 |
|  | 50-69y | 20 | 20 |
| Vitamin E (mg/day) | 18-69y | 6.5 | 6.0 |
| Vitamin K (µg/day) | 18-69y | 150 | |
| Vitamin C (mg/day) | 18-69y | 100 | |
| Vitamin B1-Thiamin (mg/day) | 18-19y | 1.4 | 1.2 |
|  | 20-29y | 1.3 | 1.1 |
|  | 30-69y | 1.2 | 1.0 |
| Vitamin B2-Riboflavin (mg/day) | 18-19y | 1.7 | 1.4 |
|  | 20-29y | 1.5 | 1.2 |
|  | 30-69y | 1.4 | 1.2 |
| Vitamin B3-Niacin (mg/day) | 18-69y | 16 | 14 |
| Vitamin B5-Acid pantothenic (mg/day) | 18-69y | 5 | |
| Vitamin B6 (mg/day) | 18-19y | 1.3 | 1.2 |
|  | 20-49y | 1.3 | 1.3 |
|  | 50-69y | 1.7 | 1.5 |
| Vitamin B7-Biotin (µg/day) | 18-19y | 25 | |
|  | 20-69y | 30 | |
| Vitamin B9-Folate (µg/day) | 18-19y | 300 | 400 |
|  | 20-69y | 400 | 400 |
| Vitamin B12 (µg/day) | 18-69y | 2.4 | |

**Supplemental Table 2s. Median intakes for minerals and vitamins by BMI status**

|  | **Total (n=317)** | **Severely underweight (n = 58)** | **Underweight (n=102)** | **Normal weight (n=157)** | **p-value^1^** |
| --- | --- | --- | --- | --- | --- |
|  | **Median (IQR)** | | | |  |
| **Minerals** |  |  |  |  |  |
| Calcium, mg | 307.2 (214.5, 402.2) | 266.6 (200.8, 409.2) | 305.1 (194.4, 402.2) | 310.0 (238.1, 393.2) | 0.40 |
| Phosphorus, mg | 976.6 (788.1, 1208.0) | 971.8 (722.1, 1207.0) | 964.6 (803.5, 1183.3) | 985.5 (802.4, 1212.7) | 0.68 |
| Magnesium, mg | 133.6 (86.0, 191.2) | 135.6 (94.2, 181.5) | 135.3 (84.3, 218.6) | 125.7 (86.0, 183.1) | 0.68 |
| Iron, mg | 8.6 (6.3, 11.4) | 7.5 (5.7, 10.3) | 8.5 (6.5, 11.3) | 9.0 (6.5, 11.7) | 0.051 |
| Zinc, mg | 7.9 (6.5, 9.8) | 8.0 (6.1, 10.0) | 7.7 (6.8, 10.0) | 8.2 (6.5, 9.6) | 0.99 |
| Selen, µg | 72.6 (50.5, 94.8) | 67.9 (49.6, 90.8) | 75.3 (50.5, 100.1) | 71.3 (51.4, 94.8) | 0.52 |
| Copper, µg | 711.9 (573.4, 981.9) | 693.5 (515.7, 935.6) | 711.6 (561.5, 1010.8) | 751.2 (579.3, 986.0) | 0.57 |
| **Vitamins** |  |  |  |  |  |
| Vitamin A, µg RAE | 1306.6 (374.0, 2363.4) | 723.1 (212.9, 1737.0) | 1296.4 (403.0, 2385.4) | 1420.8 (432.2, 2544.0) | 0.007 |
| Vitamin D, µg | 6.0 (2.1, 12.0) | 5.6 (2.0, 10.1) | 6.0 (2.2, 12.1) | 10.6 (2.1, 11.1) | 0.18 |
| Vitamin E, mg | 4.9 (2.7, 8.0) | 4.9 (2.2, 7.7) | 4.4 (2.6, 7.5) | 5.2 (3.1, 8.3) | 0.23 |
| Vitamin K, µg | 62.7 (19.6, 162.2) | 32.9 (17.7, 94.5) | 69.6 (19.8, 183.0) | 69.5 (19.9, 214.7) | 0.21 |
| Vitamin C, mg | 49.1 (22.9, 83.2) | 36.3 (18.1, 55.6) | 47.2 (23.1, 90.3) | 55.8 (25.7, 94.1) | 0.019 |
| Thiamin, mg | 0.9 (0.6, 1.2) | 0.8 (0.6, 1.0) | 0.9 (0.7, 1.2) | 0.9 (0.7, 1.3) | 0.15 |
| Riboflavin, mg | 0.4 (0.3, 0.7) | 0.4 (0.3, 0.7) | 0.5 (0.3, 0.7) | 0.5 (0.3, 0.6) | 0.76 |
| Niacin, mg | 9.5 (6.8, 13.5) | 9.3 (5.9, 10.8) | 9.9 (6.6, 13.9) | 9.5 (7.5, 14.5) | 0.29 |
| Acid pantothenic, mg | 3.1 (2.4, 4.1) | 3.0 (2.3, 3.9) | 3.1 (2.3, 4.1) | 3.1 (2.4, 4.0) | 0.66 |
| Vitamin B6, mg | 0.8 (0.5, 1.1) | 0.8 (0.5, 1.0) | 0.8 (0.6, 1.1) | 0.8 (0.5, 1.1) | 0.85 |
| Biotin, µg | 6.4 (4.6, 8.8) | 6.2 (3.8, 8.2) | 6.5 (4.6, 8.3) | 6.4 (4.6, 9.5) | 0.74 |
| Folate, µg | 115.2 (56.1, 224.2) | 98.0 (50.5, 193.8) | 113.6 (48.3, 240.5) | 117.6 (62.6, 230.6) | 0.49 |
| Vitamin B12, µg | 0.7 (0.2, 1.9) | 0.8 (0.2, 2.4) | 0.6 (0.1, 1.6) | 0.7 (0.2, 1.9) | 0.42 |

^1^p-value was calculated using one-way ANOVA tests

IQR: Interquartile range. RAE: Retinol Activity Equivalent
